# Hepatic Epstein-Barr Virus-Positive Inflammatory Follicular Dendritic Cell Sarcoma: A Diagnostic Imaging Challenge - A Focused Review and Case Report

**DOI:** 10.2174/0115734056449528260522091255

**Published:** 2026-05-25

**Authors:** Jun Guan, Yuanqing Liu, Feiwen Feng, Su Hu

**Affiliations:** 1 Department of Radiology, The First Affiliated Hospital of Soochow University, No.188 Shizi Street, Suzhou, Jiangsu Province, 215006, China; 2 Institute of Medical Imaging, Soochow University, Suzhou, Jiangsu Province, 215006, China

**Keywords:** Epstein-Barr virus-positive inflammatory follicular dendritic cell sarcoma, Inflammatory pseudotumor-like follicular dendritic cell sarcoma, Liver, Imaging findings, Computed tomography, Magnetic resonance imaging

## Abstract

**Background:**

Epstein-Barr Virus-Positive Inflammatory Follicular Dendritic Cell Sarcoma (EBV+ IFDCS) is a rare low-grade malignant neoplasm. Preoperative diagnosis of EBV+ IFDCS is challenging because it lacks specific clinical or laboratory manifestations as well as distinctive imaging features. This report presents a case of hepatic EBV+ IFDCS and reviews the relevant literature. The study summarized the imaging characteristics of this condition and provided insights to support preoperative imaging evaluation.

**Case Presentation:**

A 54-year-old asymptomatic woman was found to have a hepatic mass during a routine health screening and subsequently underwent surgical resection. Preoperative computed tomography and magnetic resonance imaging identified a lesion in the left hepatic lobe. The mass demonstrated marked but heterogeneous enhancement in the arterial phase, followed by decreased enhancement in the portal venous and delayed phases. The diagnosis of hepatic EBV+ IFDCS was confirmed by histopathological examination.

**Conclusion:**

Because the imaging findings of hepatic EBV+ IFDCS are variable, preoperative diagnosis remains challenging. This report aims to improve recognition of the imaging characteristics of this disease.

## 1 INTRODUCTION

Follicular Dendritic Cell Sarcoma (FDCS), a rare mesenchymal malignancy, was first described in 1986 [1]. Inflammatory pseudotumor-like follicular dendritic cell sarcoma (IPT-like FDCS) is a distinct subtype of FDCS, initially reported in 1996 [2] and formally named in 2001 [3]. This subtype has a strong association with Epstein-Barr virus (EBV) infection and is characterized by a prominent inflammatory infiltrate rich in lymphocytes and plasma cells. In the 5th edition of the World Health Organization Classification of Haematolymphoid Tumours, the tumor is categorized under stromal-derived tumors of lymphatic tissue and officially designated as “Epstein-Barr Virus-Positive Inflammatory Follicular Dendritic Cell Sarcoma” (EBV+ IFDCS) [4].

EBV+ IFDCS typically presents with nonspecific clinical symptoms, lacks specific tumor markers on laboratory testing, and does not demonstrate characteristic imaging features. Consequently, it is frequently misdiagnosed preoperatively as Hepatocellular Carcinoma (HCC), hepatic Inflammatory Myofibroblastic Tumor (IMT), Lymphoepithelioma-Like Intrahepatic Cholangiocarcinoma (LEICC), or other hepatic mass lesions. To date, published reports on FDCS have largely been limited to case studies or have focused mainly on pathological findings, with relatively few providing a systematic analysis of imaging characteristics.

In this report, we present a detailed case of hepatic EBV+ IFDCS confirmed by histopathology examination, with particular emphasis on its Computed Tomography (CT) and Magnetic Resonance Imaging (MRI) findings. In addition, a systematic review of previously reported cases was conducted to illustrate the clinical and imaging characteristics of this rare neoplasm. Throughout this manuscript, the terms “EBV+ IFDCS” and “IPT-like FDCS” are used interchangeably, as they appear in the original literature, with EBV status specified when available, to ensure consistency with the cited sources and facilitate readers’ interpretation of the tables and references.

## 2 CASE PRESENTATION

A 54-year-old woman was admitted after a lesion in segment IV of the liver was incidentally detected during a routine health screening. She reported no significant abdominal discomfort, fever, or weight loss. Laboratory tests were negative for Alpha-Fetoprotein (AFP), Carcinoembryonic Antigen (CEA), carbohydrate antigen 19-9 (CA19-9), and carbohydrate antigen 125 (CA125).

**Fig. (1):**
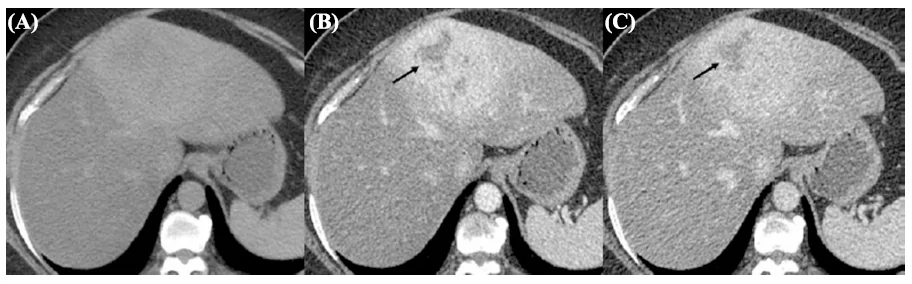
Plain and contrast-enhanced CT images. (**A**) Non-contrast CT of the abdomen showing hepatic steatosis and a mildly hypodense lesion in segment IV of the liver. (**B**) During the arterial phase, the lesion exhibited heterogeneous marked enhancement, with relatively hypodense areas within the mass. (**C**) In the portal venous phase, the enhancement decreased, and the hypodense areas observed in the arterial phase were slightly reduced in size (black arrow).

Non-contrast CT revealed a mildly hypodense mass in segment IV of the liver, measuring approximately 8.5 × 6.4 cm. On contrast-enhanced CT, the lesion demonstrated marked but heterogeneous enhancement in the arterial phase, with relatively hypodense areas within the lesion. In the portal venous phase, the degree of enhancement decreased, and the hypodense areas appeared slightly reduced in size (Fig. **1**).

**Fig. (2):**
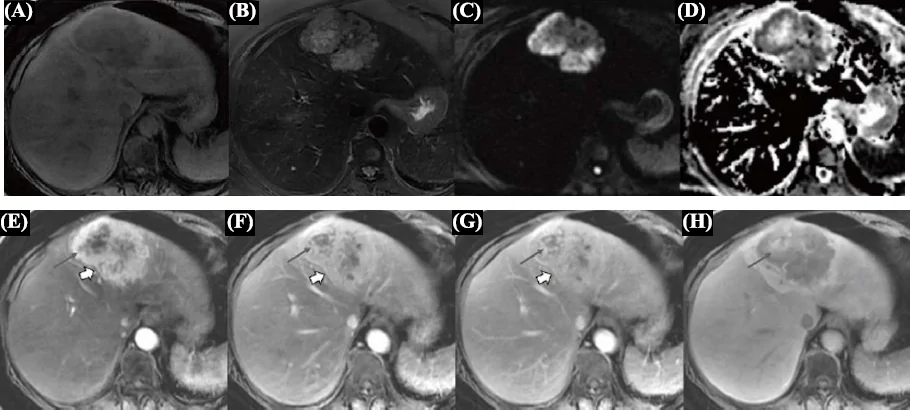
Gd-EOB-DTPA enhanced MRI images. (**A**-**D**) Non-contrast sequences: The lesion exhibited heterogeneous signal intensity, appearing mildly hypointense on T1WI, mildly hyperintense on T2WI, hyperintense on DWI, and hypointense on the ADC map. (**E**) Arterial phase: The lesion showed heterogeneous, marked enhancement. (**F**-**G**) Portal venous and delayed phases: Enhancement gradually decreased. (**H**) Hepatobiliary phase: Areas that were previously enhancing became hypointense, whereas the initially hypointense region in the arterial phase appeared hyperintense (black arrow). The lesion also contained enhancing intralesional vessels and caused displacement of adjacent vascular structures (white arrow).

Non-contrast MRI demonstrated a lesion with heterogeneous signal intensity: mildly hypointense on T1-weighted imaging (T1WI), mildly hyperintense on T2-weighted imaging (T2WI), hyperintense on diffusion-weighted imaging (DWI), and hypointense on Apparent Diffusion Coefficient (ADC) maps. Gadolinium ethoxybenzyl diethylenetriamine pentaacetic acid (Gd-EOB-DTPA)-enhanced MRI revealed prominent but heterogeneous enhancement in the arterial phase, with patchy hypointense areas within the lesion. The enhancement gradually decreased in the portal venous and delayed phases; however, the lesion remained relatively hyperintense compared with the surrounding liver parenchyma. In the hepatobiliary phase, the previously hyperenhanced areas became hypointense, while the hypointense regions observed during the early enhancement phases gradually decreased in size and appeared hyperintense. In addition, intralesional vascular structures and displacement of adjacent vessels were observed (Fig. **2**).

**Fig. (3):**
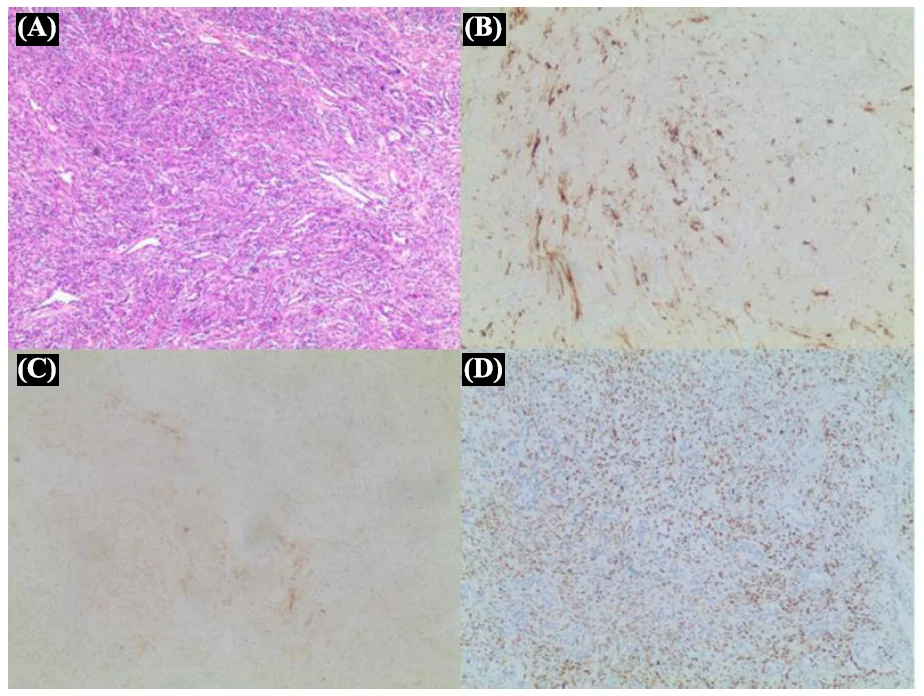
Histopathological and immunohistochemical findings of the lesion. (**A**) Hematoxylin and Eosin (H&E) staining showing spindle-shaped to oval tumor cells intermixed with abundant inflammatory cells (original magnification, 40×). (**B**) Immunohistochemical staining demonstrating focal positivity for CD21 (original magnification, 40×). (**C**) CD35 positivity observed in a subset of tumor cells (original magnification, 40×). (D) In situ hybridization for Epstein-Barr virus-encoded RNA (EBER) showing positive signals (original magnification, 40×).

The patient subsequently underwent laparoscopic left hemihepatectomy. Histopathological examination revealed scattered spindle to oval tumor cells within a background of lymphocytes and plasma cells (Fig. **3**). Immunohistochemical staining showed that a small subset of tumor cells was positive for CD35 and BCL-2, with focal positivity for CD21 and CK, while other markers, including CD23 and SMA, were negative. *In situ* hybridization detected Epstein-Barr virus-encoded RNA (EBER+) (Fig. **3**), confirming EBV infection. The final pathological diagnosis was hepatic EBV+ IFDCS. At 20 months post-surgery, there was no evidence of local recurrence or distant metastasis.

## 3 DISCUSSION

### 3.1 Clinical Overview

EBV+ IFDCS showed certain epidemiological and anatomical tendencies, occurring more frequently in female patients. The liver and spleen are the most commonly affected organs, although cases have also been reported in the colon, mesentery, palatine tonsils, and other sites [5].

Most patients with EBV+ IFDCS (or IPT-like FDCS) are either asymptomatic or present with nonspecific clinical manifestations. In some cases, symptoms such as abdominal pain, fever, weight loss, or thrombocytopenia may occur [6]. A few cases have been related to paraneoplastic syndromes, including paraneoplastic pemphigus [7-9] and paraneoplastic arthritis [10], with paraneoplastic pemphigus potentially indicating a poor prognosis [5].

The etiology and pathogenesis of FDCS are not yet fully understood. Nevertheless, substantial evidence suggests that EBV infection plays a key role in the development of EBV+ IFDCS with viral genome integration and dysregulation of the immune microenvironment, potentially contributing to tumorigenesis [11]. Additionally, FDCS may be associated with the hyaline-vascular subtype of Castleman disease [12, 13] and with IgG4-related disease [14, 15]. EBV-negative cases have also been reported in recent years [8, 16-18], indicating the existence of alternative pathogenic mechanisms.

### 3.2 Imaging Findings

To better characterize the imaging features of this tumor, we conducted a systematic review of the literature published over the past 10 years (up to May 31, 2025) using the PubMed database. The search terms included: “inflammatory pseudotumor-like follicular dendritic cell sarcoma,” “Epstein-Barr virus-positive inflammatory follicular dendritic cell sarcoma,” “EBV+ IFDCS”, and “IPT-like FDCS.” The inclusion criteria were: (1) histopathologically confirmed diagnosis of hepatic IPT-like FDCS or EBV+ IFDCS; and (2) availability of a description of CT and/or MRI findings. The exclusion criteria were: (1) cases in which the imaging description did not include contrast-enhanced findings, and (2) cases where imaging findings could not be attributed to a specific modality. Including the present case, a total of 24 cases were identified. CT findings are summarized in Table **1**, and MRI findings in Table **2**. Additionally, two cases of EBV-negative IPT-like FDCS [17, 18] were included in the discussion. For Case 11 in Table **1** and Case 3 in Table **2**, the description of marked enhancement in the arterial phase was inferred from both the text and imaging figures in the original publication.

#### 3.2.1 Imaging Findings on Non-contrast CT and MRI

On non-contrast CT, hepatic lesions are typically hypodense relative to the surrounding liver parenchyma. On MRI, they generally appear hypointense on T1WI and hyperintense on T2WI. Larger lesions are more likely to undergo necrosis or cystic degeneration, and calcification has been reported in a few cases. DWI often shows high signal intensity, reflecting restricted water diffusion due to the high cellular density of the tumor [32].

#### 3.2.2 Enhancement Patterns

The enhancement patterns of the tumor were heterogeneous and classified as follows: (1) Early Marked Enhancement: In 33.3% (8/24) of cases, the lesion exhibited marked enhancement during the arterial phase, with enhancing intratumoral vessels observed in some cases. Most of these lesions showed varying degrees of decreased enhancement in the portal venous or delayed phases. One case, in particular, demonstrated an enhancement pattern described as “rapid wash-in and slow wash-out” [26]. (2) Progressive or Delayed Enhancement: Several lesions showed overall progressive or delayed enhancement. (3) Other Enhancement Patterns: Some lesions exhibited other patterns, including mild overall enhancement, persistent hypoenhancement, and rim enhancement. These varied enhancement patterns reflect the complex intratumoral composition, including the distribution of tumor cells, microvasculature, inflammatory cells, fibrous tissue, and areas of necrosis. The associations between enhancement patterns and tumor components need further investigation with larger sample sizes and histopathological validation in future studies.

#### 3.2.3 Other Imaging Features

In some cases, areas that appeared hypointense or hypodense during the arterial phase demonstrated enhancement in subsequent phases, accompanied by a reduction in their size. This phenomenon may be attributed to two factors: first, these regions may represent areas of incomplete coagulative necrosis containing fibrous components and small vessels [26]; second, it may reflect the relative proportions of tumor cells and fibrous tissue within these regions [24]. Similar imaging findings have been reported in splenic IPT-like FDCS [33]. Owing to the longer acquisition times of MRI, this phenomenon is more frequently observed on MRI examinations [26].

In four cases, the presence of a capsule or pseudocapsule was clearly observed. Composed mainly of fibrous tissue, the capsule typically appeared hypointense on T2WI [21]. Capsules containing small vessels often demonstrated delayed enhancement. A “halo sign” surrounding two hepatic EBV+ IFDCS lesions on T2WI has been described [21]. This sign was pathologically confirmed to be associated with an incomplete tumor capsule with peritumoral edema and inflammatory cell infiltration. In the review of 24 cases, this finding was observed only in these two cases and was not consistently reported in others, suggesting that the “halo sign” may not be a characteristic feature of this rare tumor. Nevertheless, when present in an atypical hepatic lesion, this sign may broaden the differential diagnosis and prompt consideration of EBV+ IFDCS.

In three cases, patchy hyperintense areas were observed during the hepatobiliary phase. The exact mechanism underlying this finding remains unclear. This may be related to the expression of membrane transport proteins such as OATP1B1/3 and MRP3 [34], or may result from retention of contrast agents within mucin-rich stromal regions [32].

In one case, abnormal perfusion was observed around the lesion, potentially resulting from tumor invasion of the hepatic venous system, causing arteriovenous fistulas, or from compression of the portal vein by the tumor, leading to a compensatory increase in arterial blood flow [35].

#### 3.2.4 Differential Diagnosis

Hepatic EBV+ IFDCS should be differentiated from the following conditions. (1) HCC: HCC commonly arises in the context of chronic liver disease and is often associated with increased AFP levels. It typically exhibits a “rapid wash-in and rapid wash-out” enhancement pattern, whereas EBV+ IFDCS may show a “rapid wash-in and slow wash-out” pattern, which can aid in differentiation. (2) IMT: IMT generally demonstrates minimal enhancement during the arterial phase. Enhancement patterns in the portal venous and delayed phases are variable, but a relatively distinctive feature is the presence of penetrating portal vein branches within the lesion [36]. (3) LEICC: LEICC is associated with EBV infection and typically shows arterial phase hyperenhancement followed by gradual wash-out in the portal venous and delayed phases. The presence of central delayed enhancement may aid in differential diagnosis [37]. (4) Perivascular Epithelioid Cell Tumor (PEComa): PEComa commonly exhibits “rapid wash-in and rapid wash-out” or “rapid wash-in and slow wash-out” enhancement patterns [38]. The presence of large, aberrant vessels and intralesional fat components can help distinguish PEComa from other hepatic lesions [39]. (5) Intrahepatic Cholangiocarcinoma (ICC): ICC is typically EBV-negative and is often associated with increased serum CA19-9 levels. Common imaging features include capsular retraction and biliary dilatation [40]. Underlying risk factors such as primary sclerosing cholangitis are frequently present. In contrast, these clinical, serological, and imaging characteristics are rarely observed in cases of EBV+ IFDCS.

We summarized several imaging characteristics of hepatic EBV+ IFDCS. However, because only cases with detailed imaging descriptions were included, there is an inherent selection bias. Larger studies are needed to more accurately define the characteristic imaging features of hepatic EBV+ IFDCS.

### 3.3 Treatment and Prognosis

Radical surgical resection remains the primary treatment for FDCS [41]. In cases with extensive hepatic involvement, trans-arterial chemoembolization has been used effectively for preoperative downstaging [42]. The role of postoperative adjuvant therapy remains controversial. Some studies indicate that radiotherapy and chemotherapy do not significantly improve overall or disease-free survival [43, 44]. However, in patients with advanced disease or incompletely resected tumors, radiotherapy and chemotherapy may provide meaningful tumor control [45]. Accordingly, treatment strategies for FDCS should be individualized, taking into account the patient's condition, tumor characteristics, and other relevant factors.

Immune checkpoint inhibitors targeting PD-1/PD-L1 have shown potential therapeutic value and promising efficacy in clinical trials for EBV-associated tumors [46]. Although a high rate of PD-L1 positivity has been reported in EBV+ IFDCS, indicating its potential as an immunotherapy target, PD-L1 expression levels are heterogeneous [47]. Therefore, the efficacy of anti-PD-1/PD-L1 immunotherapy in FDCS requires further validation in large studies.

EBV+ IFDCS generally exhibits lower metastatic potential compared with classic FDCS [48]. A study of 92 patients with IPT-like FDCS reported a recurrence or metastasis rate of 9.65% over a follow-up period of 2 to 144 months [30]. In an analysis of 44 patients with hepatic IPT-like FDCS, 15.9% developed metastases, all of whom had primary tumors measuring ≥6 cm [6]. Tumor size (≥6 cm), a high mitotic rate (≥5 per 10 high-power fields), necrosis, and marked cytological atypia are associated with a more aggressive tumor phenotype [49]. Notably, different studies have proposed varying tumor size thresholds as prognostic indicators. Another study suggested that hepatic location, patient age under 41 years, and a maximum tumor diameter greater than 9 cm may serve as adverse prognostic factors for IPT-like FDCS [50]. The differing tumor size thresholds reported in previous studies likely reflect heterogeneity in study populations. While tumor size is an important prognostic indicator, it represents only one of several factors influencing outcomes. In our case, the tumor measured over 6 cm, yet no recurrence was observed at 20 months, highlighting the need to interpret tumor size alongside other pathological and clinical features.

## CONCLUSION

In summary, we present a new case and a literature review to provide guidance for the preoperative diagnosis of hepatic EBV+ IFDCS. Our findings indicate that a comprehensive analysis of diverse enhancement patterns and suggestive imaging features, such as the “halo sign”, combined with the clinical context of no underlying liver disease and normal AFP levels, can aid in diagnosis.

## Figures and Tables

**Table 1:** CT imaging findings of hepatic EBV+ IFDCS and IPT-like FDCS.

**Case/Refs.**	**Age(y)/Sex**	**Non-contrast CT**	**Enhancement Pattern**	**Other Imaging Findings**
1 [19]	54/F	NA	Enh. (AP); Subsequent washout (phase not specified)	Capsule
2* [17]	70/F	NA	Marked Enh. (AP); Isodense to liver (VP)	NA
3 [20]	23/F	NA	Marked Enh. (AP); Decreased Enh. (VP)	Pseudocapsule
4 [21]	64/M	Mildly hypodense	Mild Enh. (AP); Decreased Enh. (PVP and DP)	NA
5 [21]	57/F	Hypodense	Grid Enh.	NA
6 [22]	31/F	NA	Heterogeneous Enh. (AP); No significant washout (PVP)	NA
7 [23]	34/F	Hypodensity	Persistent hypoenhancement (AP, VP, and DP)	Peripheral abnormal perfusion
8 [18]	61/M	Heterogeneous density	Hypoenhancement (AP); Decreased Enh. (PVP and DP)	NA
9* [24]	31/F	Hypodensity	Isoenhancement (AP); Hypoenhancement (PVP and DP)	Initial hypodense areas decreased in size
10 [24]	48/M	Hypodensity	Persistent hypoenhancement (AP, PVP, and DP)	Distorted blood vessels
11 [25]	60/F	NA	Marked Enh. (AP); Isodense to liver (DP)	NA
12* [26]	16/M	NA	Heterogeneous fill-in Enh.	Capsule
13* [26]	NA	Isodensity	Marked Enh. (AP); Decreased Enh. (VP and DP); Mild hyperdense to liver (DP)	NA
14* [27]	45/M	Heterogeneous density	Progressive Enh.	Calcification
15 [28]	38/M	Heterogeneous density	Heterogeneous Enh. (AP); Decreased Enh. (PVP)	Large feeding arteries
Present case*	54/F	Mildly hypodensity	Marked Enh. (AP); Decreased Enh. (PVP)	Initial hypodense areas decreased in size

**Abbreviations:** F = female; M = male; NA = not available; AP = arterial phase; VP = venous phase; PVP = portal venous phase; DP = delayed phase; Enh. = enhancement; CT = computed tomography; MRI = magnetic resonance imaging. Superscript letters * indicate cases with both CT and MRI imaging findings.

**Table 2:** MRI imaging findings of hepatic EBV+ IFDCS and IPT-like FDCS.

**Case/Refs.**	**Age(y)/Sex**	**T1WI**	**T2WI**	**DWI**	**Enhancement Pattern**	**Other Imaging Findings**
1* [17]	70/F	NA	Hyper.	NA	Marked Enh. (AP); Washout (VP)	Hypo.(excretory phase)
2 [6]	55/F	Heterogeneous hypo.	Mildly hyper.	Hyper.	Peripheral Enh. (AP); Mild internal delayed Enh. (PVP and DP)	NA
3 [29]	35/F	NA	NA	NA	Marked Enh. (AP); Washout (PVP)	NA
4 [30]	66/F	Hypo.	Mildly hyper.	Hyper.	Progressive Enh. (AP, PVP, and DP)	NA
5 [21]	46/M	Mildly hypo.	Mildly hyper.	Hyper.	Marked central Enh. (AP); Progressive Enh.(VP); Decreased Enh. with rim Enh (DP)	Presence of a “halo sign”; Centrifugal Enh.
6 [31]	67/M	Mildly hypo.	Mildly hyper.	Mildly hyper.	Mild circular Enh.	NA
7* [24]	31/M	Hypo.	Hyper.	NA	Persistent hypoenhancement (AP, PVP, and DP)	Patchy Hyper.(HBP); Initial hypointense areas decreased in size
8* [26]	16/M	Mildly hypo.	Hyper.	Hyper.	Mild Enh. (AP); Progressive fill-in Enh. (PVP); Persistent Enh. (DP)	Delayed capsular Enh; Initial hypointense areas decreased in size
9* [26]	NA	NA	Mildly hyper.	Hyper.	Marked Enh. (AP); Decreased Enh. (PVP and DP); Mildly hyper. (DP)	NA
10 [26]	NA	NA	Mildly hyper.	Hyper.	Heterogeneous fill-in Enh.	NA
11* [27]	45/M	Heterogeneous intensity	Hyper.	Hyper.	Progressive Enh.	NA
12 [32]	53/M	Mildly hypo.	Mildly hyper.	Hyper.	Marked Enh. (AP); Isointense to liver (PVP); Hypointense to the liver (TP)	Patchy hyper. (HBP); Initial hypointense areas decreased in size; Tumor vessels; Pseudocapsule
13 [23]	40/F	NA	Hyper.	Hyper.	Delayed Enh.	NA
Present case*	54/F	Mildly hypo.	Mildly hyper.	Hyper.	Marked Enh. (AP); Decreased Enh. (PVP and DP)	Patchy Hyper. (HBP); Initial hypointense areas decreased in size; Tumor vessels;

**Abbreviations:** F = Female; M = Male; NA = Not Available; T1WI = T1-Weighted Imaging; T2WI = T2-Weighted Imaging; DWI = Diffusion-Weighted Imaging; AP = Arterial Phase; VP = Venous Phase; PVP = Portal Venous Phase; DP = Delayed Phase; TP = Transitional Phase; HBP = Hepatobiliary Phase; Enh. = Enhancement; Hyper. = Hyperintense; Hypo. = Hypointense; CT = Computed Tomography; MRI = Magnetic Resonance Imaging. Superscript letters * indicate cases with both CT and MRI imaging findings.

## Data Availability

All data generated or analyzed during this study are included in this published article.

## LIST OF ABBREVIATIONS

ADC: Apparent diffusion coefficient
AFP: Alpha-fetoprotein
CA125: Carbohydrate antigen 125
CA19-9: Carbohydrate antigen 19-9
CEA: Carcinoembryonic antigen
CT: Computed tomography
DWI: Diffusion-weighted imaging
EBER: Epstein-Barr virus-encoded RNA
EBV: Epstein-Barr virus
EBV+ IFDCS: Epstein-Barr virus-positive inflammatory follicular dendritic cell sarcoma
FDCS: Follicular dendritic cell sarcoma
Gd-EOB-DTPA: Gadolinium ethoxybenzyl diethylenetriamine pentaacetic acid
HCC: Hepatocellular carcinoma
ICC: Intrahepatic cholangiocarcinoma
IMT: Inflammatory myofibroblastic tumor
IPT-like FDCS: Inflammatory pseudotumor-like follicular dendritic cell sarcoma
LEICC: Lymphoepithelioma-like intrahepatic cholangiocarcinoma
MRI: Magnetic resonance imaging
PEComa: Perivascular epithelioid cell tumor
T1WI: T1-weighted imaging
T2WI: T2-weighted imaging

## STANDARDS OF REPORTING

CARE guidelines were followed.
